# The Relation of the School Doctor to the Private Practitioner

**Published:** 1914-07-18

**Authors:** 


### THE SCHOOL DOCTOR.

### The Relation of the School Doctor to the Private Practitioner.

Recently the extension of the school clinic has brought the school doctor into somewhat more intimate relation with the private practitioner. Where the school treatment centre is under the local authority and staffed by whole-time men, there is, of course, comparatively little possibility of friction. But unfortunately such a state of things is not yet general. Many local authorities already possess a clinic of their own, but the majority still seek the aid of voluntary effort in coping with the mass of defective children. In such cases the general rule is that the local practitioners form a private panel; they rent a set of rooms or a building, equip it for purposes of a school clinic, and get, in return for the services that they render, a grant in aid from the local authority. The staffing and professional control of the clinic are, therefore, in their own hands. From among their number they select one or two men who take up a speciality. One, for example, does eyes, another devotes himself to nose and throat work, and a third deals with minor ailments. Where the local panel is enthusiastic and keen, the work done at these clinics is often of a very high order, fully comparable to what is achieved in the best hospital. But sometimes the reverse is the case. The panel members regard the clinic as a necessary evil, and devote only a small portion of their energies to it; they appear to think that its establishment is necessary to keep the practice among school children in their own hands, and they rather resent suggestions from the school doctor, even though these may be offered with the best intentions in the world. It is in such circumstances that the need for tact and diplomacy on the part of the school doctor in his relations with his fellow-practitioners becomes apparent.

### The Importance of the Follow Up.

Cases sent to a school clinic must be followed up. It implies no suspicion of the methods of the staff at such a clinic, nor any aspersion upon their professional capabilities, for the school doctor to review from time to time the results of their work. In many cases he is a newcomer in the district, and unacquainted with the practitioners, but his experience will enable him to form an opinion of the work done. If cases are followed up regularly some check is secured; cases not treated or badly treated can be sent back to the clinic, and consultative action may be taken in regard to parents who have refused treatment. Nor must it be assumed that badly treated cases necessarily mean bad treatment on the part of the clinic staff. For instance, a case of adenoids may, on being followed up, be noted as "badly treated" in so far as proper nasal breathing has not been re-established. This means, generally, one of two things, either the operation has not been properly performed or the after-treatment has been neglected. So far as the former is concerned, no opinion can be given from a mere examination such as in general is deemed sufficient when a case is followed up; to find out whether the operation has been properly performed, a post-nasal examination has to be undertaken. Generally the fault lies in the after-treatment, in so far that the patient has neglected to observe the directions given for breathing exercises. If such a patient is referred back to the clinic the operator has a chance of noting the case and of repeating his instructions, and if the reference back is tactfully done by the school doctor, no one's feelings need be hurt.

Where friction does ensue, in the case of clinic work, it is because of the neglect on the part of one or other of the parties concerned to bear in mind the elementary rules of professional courtesy. Neither the school doctor nor any member of the clinic staff should express any opinion on the merits of each other's work in the hearing of a parent or child. For the clinic doctor to tell a parent who comes to him with the report of the school doctor that "there is nothing whatever the matter with your child," is as bad as for the school doctor to tell the parent at a following-up examination "Your child has been badly treated." We are all liable to make mistakes, and neither school doctors nor members of a school treatment centre are infallible. But the school doctor generally has some grounds for his report to the parent, and the clinic practitioner nearly always does his work to the best of his skill and ability. Tact and courtesy will bridge the widest gap that looms between the two, and when these are employed as a matter of course between school doctor and clinic practitioners, their relationship will never be strained.

From the school doctor's point of view, cordial co-operation with the practitioners on the clinic panel is highly desirable. They are for the most part men in active medical practice in the neighbourhood of the schools where his work lies; they can, therefore, indirectly assist him most materially in his labours so far as the children are concerned. Moreover, they are generally intimately acquainted with the economic conditions of the community; they know what parents cannot afford treatment, and what parents can afford to go to their own doctor for treatment or instruments. It is to the school doctor's advantage to have periodical consultations with them. Indeed, the establishment of school clinics in which local practitioners take an active interest is bound, in course of time, to react advantageously upon the profession. Such clinics give the local practitioner the means of perfecting himself in certain specialities in which he may be interested—a means which, unless he has the good fortune to be on a hospital staff (and in the case of large towns it is impossible for the private practitioner to aspire to a hospital post), he has hitherto lacked.

